# Selective binocular vision loss in two subterranean caviomorph rodents: *Spalacopus cyanus* and *Ctenomys talarum*

**DOI:** 10.1038/srep41704

**Published:** 2017-02-02

**Authors:** T. Vega-Zuniga, F. S. Medina, G. Marín, J. C. Letelier, A. G. Palacios, P. Němec, C. E. Schleich, J. Mpodozis

**Affiliations:** 1 Departamento de Biología, Facultad de Ciencias, Universidad de Chile, Santiago, de Chile; 2 Lehrstuhl für Zoologie, Technische Universität München, Freising-Weihenstephan, Germany; 3 Facultad de Medicina, Universidad Finis Terrae, Santiago, Chile; 4 Centro Interdisciplinario de Neurociencia de Valparaíso, Facultad de Ciencias, Universidad de Valparaíso, Valparaíso, Chile; 5 Department of Zoology, Faculty of Science, Charles University in Prague, Prague, Czech Republic; 6 Laboratorio de Ecofisiología, Instituto de Investigaciones Marinas y Costeras, Universidad Nacional de Mar del Plata, Mar del Plata, Argentina

## Abstract

To what extent can the mammalian visual system be shaped by visual behavior? Here we analyze the shape of the visual fields, the densities and distribution of cells in the retinal ganglion-cell layer and the organization of the visual projections in two species of facultative non-strictly subterranean rodents, *Spalacopus cyanus* and *Ctenomys talarum*, aiming to compare these traits with those of phylogenetically closely related species possessing contrasting diurnal/nocturnal visual habits. *S. cyanus* shows a definite zone of frontal binocular overlap and a corresponding area centralis, but a highly reduced amount of ipsilateral retinal projections. The situation in *C. talarum* is more extreme as it lacks of a fronto-ventral area of binocular superposition, has no recognizable area centralis and shows no ipsilateral retinal projections except to the suprachiasmatic nucleus. In both species, the extension of the monocular visual field and of the dorsal region of binocular overlap as well as the whole set of contralateral visual projections, appear well-developed. We conclude that these subterranean rodents exhibit, paradoxically, diurnal instead of nocturnal visual specializations, but at the same time suffer a specific regression of the anatomical substrate for stereopsis. We discuss these findings in light of the visual ecology of subterranean lifestyles.

It is generally accepted that subterranean environments favor magnetic, sound, tactile, vibratory and olfactory cues to the detriment of visual ones^1^,^2^. Nevertheless, the cumulative body of anatomical and behavioral evidence suggests that even in strictly subterranean mammals, light perception and low acuity vision play indispensable roles in photic entrainment of activity rhythms, photic control of seasonal reproduction, predator avoidance and tunnel maintenance^3^,^4^,^5^,^6^. Subterranean mammals show a great diversity of ocular arrangements, including variations in eye and corneal size, retinal thickness, and number/distribution of ganglion cells^2^,^7^,^8^,^9^,^10^. In addition, though less extensively studied, the central visual nuclei follow similar trends, showing varying levels of regression in different subterranean species^11^,^12^,^13^,^14^. For example, the blind mole-rat *Spalax ehrenbergi* has very small subcutaneous eyes with highly reduced numbers of ganglion cells and vestigial visual nuclei, except for the suprachiasmatic nucleus (SCN)^13^. On the other hand, the northern mole-vole *Ellobius talpinus* has normal sized eyes with a moderately reduced number of retinal ganglion cells and fairly well-developed central visual nuclei^14^.

Moreover, the degree of visual system reduction seems to correlate with the fraction of daily time spent underground^1^,^2^. Strictly subterranean species, such as the blind mole-rat, which live almost exclusively underground, show highly regressive visual systems, whereas facultative non-strictly subterranean species, such as the mole-vole, the coruro (*Spalacopus cyanus*) and the tuco-tucos (Ctenomyidae, Rodentia) which spend a significant fraction of time on above-ground activities, are known for having less regressive visual systems^9^,^10^.

In the case of facultative non-strictly subterranean species, no study so far has considered whether and to what extent underground behavior affects different features of the visual system, such as visual field organization, retinal specializations and central projections. We recently showed that such suit of visual traits can indeed be differentially shaped by different visual lifestyles, even between phylogenetically close related species living in contrasting visual ecotopes. Specifically, we found that binocular and not monocular components of the visual system in the nocturnal *O. lunatus* appear enhanced with respect to its diurnal sister species *O. degus*^15^, suggesting that the adoption of nocturnal habits may lead to an enhancement of the binocular visual operations.

Given that visual conditions inside burrows might be similar to those of nocturnal environments, the aforementioned results would allow to expect the presence of binocular, nocturnal-like specialization in the visual features of subterranean species. However, visual conditions faced by nocturnal animals may well greatly differ from those encountered by subterranean animals: on the one hand the burrowing environment seems not to be scotopic but almost lightless; on the other, facultative non-strictly subterranean species behave in a dual fashion between the underground ecotope and the photopic conditions around burrow entrances^1^,^2^. In this regard, interestingly most studies so far have shown that facultative non-strictly subterranean animals exhibit retinal features more close to diurnal than to nocturnal mammals (see discussion).

These previous observations prompted us to ask in which way the visual conditions enacted by the subterranean lifestyle have shaped the visual system organization in the facultative non-strictly subterranean animals. A straightforward way to address this question is to compare the visual features of subterranean animals with those of phylogenetically closely related species exhibiting different visual habits (e.g.: diurnal/nocturnal). Quite notably, besides the diurnal and nocturnal species previously studied by us, the Octodontoidea superfamily^16^ includes several subterranean members, thus allowing direct comparisons between them.

In this study, we analyzed the main structural features associated with binocular and monocular vision in two related species that have independently adopted the underground lifestyle: *Spalacopus cyanus* and *Ctenomys talarum*, and compared them with those phylogenetically related octodontids that exhibit diurnal and nocturnal visual habits (see above). In particular, we investigated the extent of the visual field, the distribution and densities of cells in the retinal ganglion cell layer, and the characteristics of retinal central projections. The results indicate that facultative non-strictly subterranean visual habits correlate with an unexpected regression of the visual traits underlying binocular fusion. We discuss these results in the context of the visual ecology of subterranean animals.

## Results

### Visual field measurements

**Figure 1:**
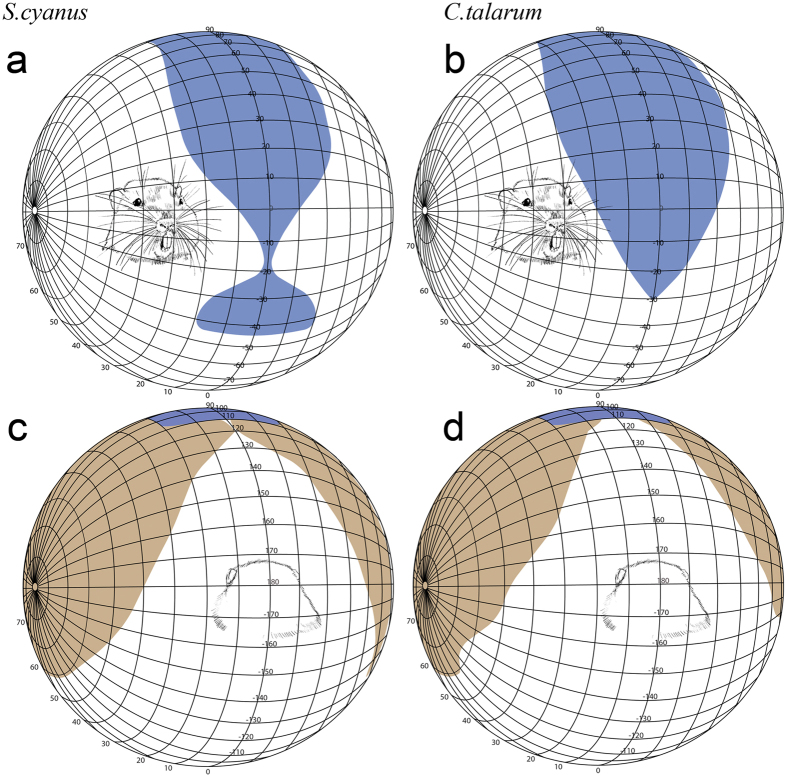
(**a–c**) Perspective views of orthographic projections of the frontal binocular (blue, **a** and **b**) and caudal monocular (brown, **c** and **d**) visual fields in the subterranean *S. cyanus* and *C. talarum*. In both rodents the maximum binocular overlap is located between 25–50° in elevation (**a**,**b**). Note the bottleneck between −10° and −20° of the binocular overlap in *S. cyanus* resulting from the nose protruding into the binocular field (**a**). Interestingly, this bottleneck shape of the binocular field is absent in *C. talarum* (**b**). The diagrams use the conventional latitude and longitude system. The drawings of a representative subterranean rodent inside the orthographic projections, courtesy of Ricardo Di Parodi.

Since conjugated convergent or divergent eye movements can greatly increase or decrease the extent of binocular overlap depending on the specific animal requirements, most studies (including this one) report measurements of the relaxed akinetic position of the eyes. We show the results obtained for one representative animal for each species (Fig. 1a,b).

The monocular visual field of *S. cyanus* had a maximum azimuthal extension of 170° that spanned from −25° frontally to 145° caudally (Fig. 1c). The region of maximum binocular overlap covered 50° and it was located dorso-rostrally with respect to the visual field, between 30° and 60° of vertical elevation (Fig. 1a). Towards the dorso-caudal portion of the visual field, the binocular overlap covered 40° and extended to 110° on the vertical axis (Fig. 1c). The rostro-ventral portion of the binocular overlap showed a characteristic bottleneck shape between −10° and −20° (vertical axis) that corresponded to the projection of the snout tip (Fig. 1a). After this narrowing, the binocular overlap continued ventrally and increased up to 40° of overlap between −30° and −45° on the vertical axis. In *C. talarum*, the monocular visual field had a maximum azimuthal extension of 170°, spanning from −25° frontally to 145° caudally (Fig. 1d). The region of maximum binocular overlap covered 60° (Fig. 1b). The location was dorso-rostral with respect to the visual field, between 30° and 60° of vertical elevation. Towards the dorso-caudal portion of the visual field, the binocular overlap covered 40° and extended to 110° on the vertical axis (Fig. 1d). The rostro-ventral overlap showed the shape of an inverted triangle, whose inferior vertex was located at −30° on the vertical axis (Fig. 1b). Interestingly, below this point no fronto-ventral binocular expansion was found.

### Distribution and density of cells in the ganglion cell layer

**Figure 2:**
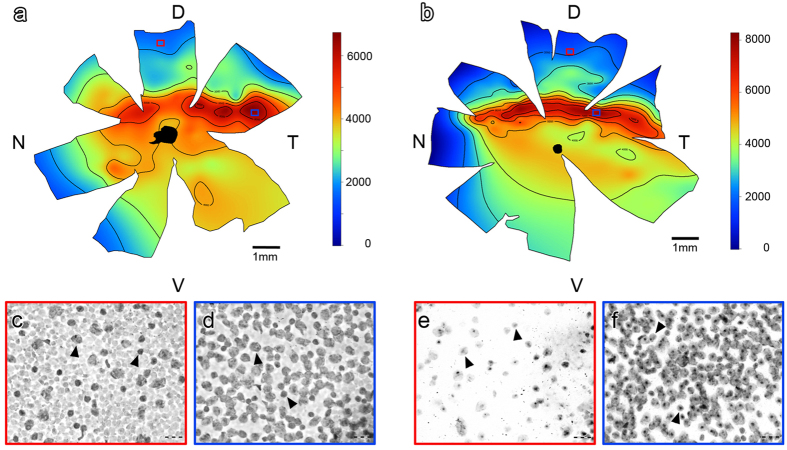
Retinal whole mounts. Topographic isodensity map reconstructions of cells in the retinal ganglion cell layer (GCL) of *S. cyanus* (**a**) and *C. talarum* (**b**). The black area in the central retina indicates the optic nerve head. Note that the decline in cell density is more pronounced towards the dorsal retina. (**c**–**f**) Insets with red and blue contours showing photomicrographs of Nissl-stained regions of the dorsal and temporal retina, respectively, as indicated in the wholemounts. Arrowheads indicate neurons in the ganglion cell layer. D = dorsal; N = nasal; T = temporal; V = ventral. Scale bar insets = 20 μm.

Retinal whole mounts were prepared from one eye of each of the individuals. We found only minor differences between individuals from the same species. Due to that, here we only show data for representative cases (Fig. 2). *S. cyanus* had a retinal area of 42 mm^2^, and an estimated total number of 139,893 cells in the ganglion cell layer. For *C. talarum* the retinal area was 50 mm^2^, and the estimated total cell number was 170,803. The mean cell density (*cells*/*retinal area*) was 3,321/mm^2^ for *S. cyanus* and 3,407/mm^2^ for *C. talarum* (Table 1). The distribution of these cells was not homogeneous. *S. cyanus and C.talarum*’s retinal density map showed a well-developed naso-temporal visual streak that runs above the optic nerve head. In both species, cell density decreases abruptly towards the dorsal regions of the retina (as shown in Fig. 2). Remarkably, we found a circumscribed higher cell density region or area centralis (AC), only in *S. cyanus* (Fig. 2a). The peak density in the AC was 6,400 cells/mm^2^. On the other hand, *C. talarum* showed no presence of an AC, although we observed a high cell density visual streak with a maximum isodensity curve of 7,744 cells/mm^2^ that extended naso-temporally (Fig. 2b, Table 1).

### Central visual projections

**Figure 3:**
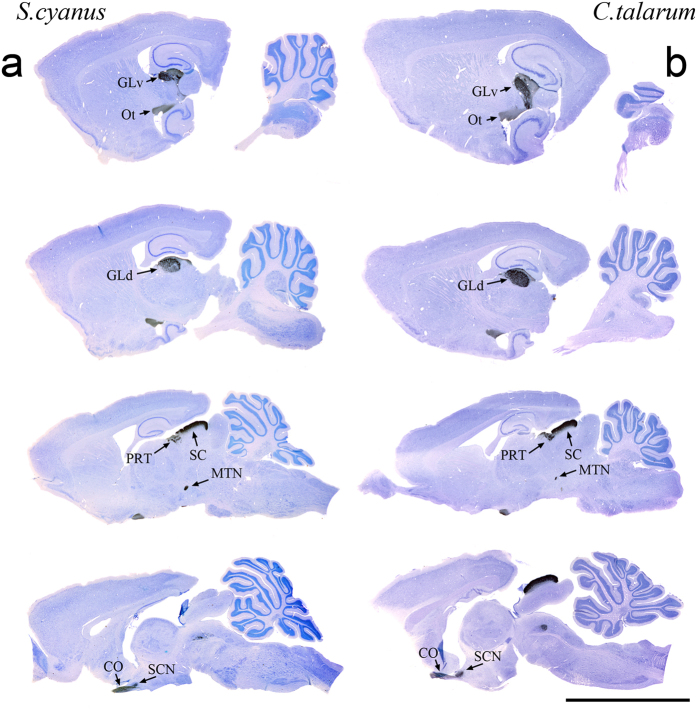
Contralateral sagittal sections showing CTB-labeled retinal fibers counterstained with Giemsa in *S. cyanus* and *C. talarum* (**a**,**b**). N. geniculatus lateralis pars ventralis (GLv), n. geniculatus lateralis pars dorsalis (GLd), superior colliculus (SC), pretectum (PRT), medial terminal nucleus (MTN), suprachiasmatic nucleus (SCN), optic tract (Ot), optic chiasma (CO). Rostral is to the left. Scale bar = 0.8 cm.

In both species we found that all major visual projections were easily recognizable; namely, the superior colliculus (SC), n. geniculatus lateralis pars dorsalis (GLd), n. geniculatus lateralis pars ventralis (GLv), suprachiasmatic nucleus (SCN), pretectal complex (PRT), and accessory optic system (AOS, e.g., medial terminal nucleus (MTN)) (Fig. 3). Contralateral visual projections were observed in all aforementioned structures, whereas ipsilateral projections were observed only in the SC, GLd, GLv and SCN of *S. cyanus* and in the SCN of *C. talarum*.

### N. geniculatus lateralis pars dorsalis (GLd)

The GLd of rodents has been characterized as a cytoarchitectonically homogeneous structure with no obvious lamination. However, previous observations in the hooded rat (*Rattus norvegicus*) indicate the presence of a *hidden* tri-laminar organization evidenced mainly by a differential density of retinal terminals^17^,^18^. Our data showed, in both species, minor differences between a narrow external (dorsal) lamina containing coarse retinal terminals, and a broader internal lamina, containing slightly less dense, finer retinal terminals. In both species the external lamina appeared as a continuum of labeled fibers that covered the whole dorsal extension of the nucleus. These labeled fibers were evident only at the contralateral side. The ipsilateral external lamina appeared free of retinal terminals.

**Figure 4:**
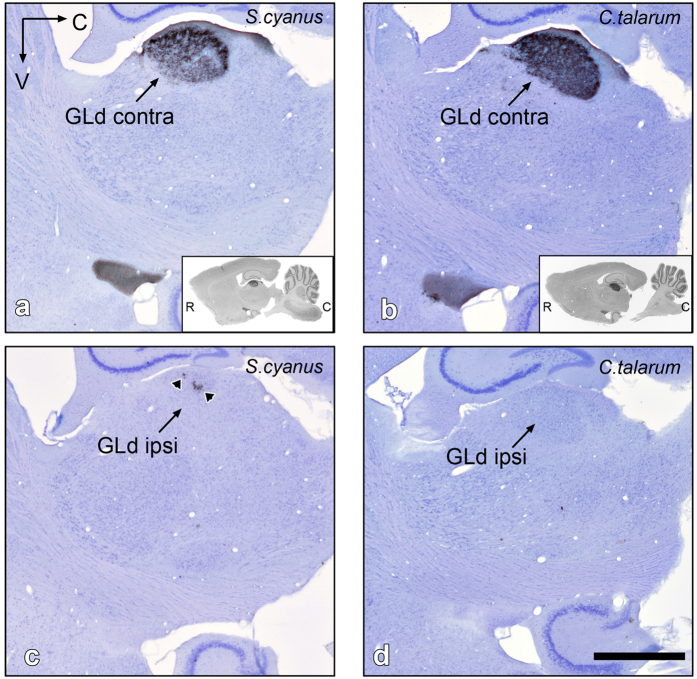
Sagittal sections showing CTB-labeled retinal terminals in the contra- and ipsilateral GLd of *S. cyanus* (**a**,**c**) and *C. talarum* (**b**,**d**). Arrowheads in (**c**) indicate ipsilateral terminals in the GLd of *S. cyanus*. Note that in (d) there are no retinal fibers in the ipsilateral GLd of *C. talarum*. Insets in (**a**,**b**) represent low magnification photomicrographs of the respective sagittal GLd. Counterstaining with Giemsa. R = rostral, C = caudal, V = ventral. Scale bar = 1 mm.

**Figure 5:**
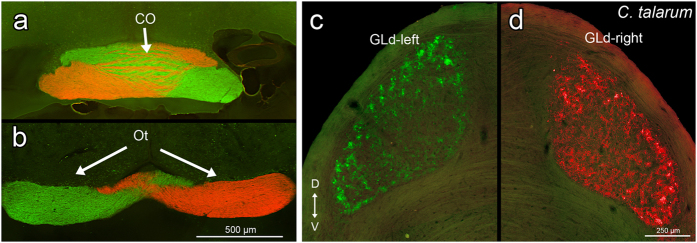
Transverse sections showing CTB-Alexa (488 and 546) labeled retinal fibers in the optic chiasma (CO) and optic tract (Ot) (**a**,**b**). Left (CTb-Alexa 488) and right (CTb-Alexa 546) labeled retinal terminals in the n. geniculatus lateralis pars dorsalis (GLd) of *C. talarum* (**c**,**d**). Note that there is no labeling of ipsilateral fibers in the GLd. Orientation in (**c**) applies to all sections. D = dorsal; V = ventral.

In *S. cyanus* the internal lamina showed at the contralateral side a clear small band lacking labeled retinal fibers (as shown in Fig. 4a). This zone was located in the centro-caudal third portion of the nucleus. On the other hand, most of the ipsilateral side appears free of labeling, with the exception of a small patchy band of terminals centro-caudally located within the internal lamina (as shown in Fig. 4c). This patchy area corresponds in shape and position to the empty contralateral side band. Most interestingly, in *C. talarum* the contralateral GLd showed no band lacking of labeled fibers (Figs 4b and 5c,d), and in the ipsilateral GLd, there was no evidence of retinal projections (Figs 4d and 5c,d).

### Superior colliculus (SC)

**Figure 6:**
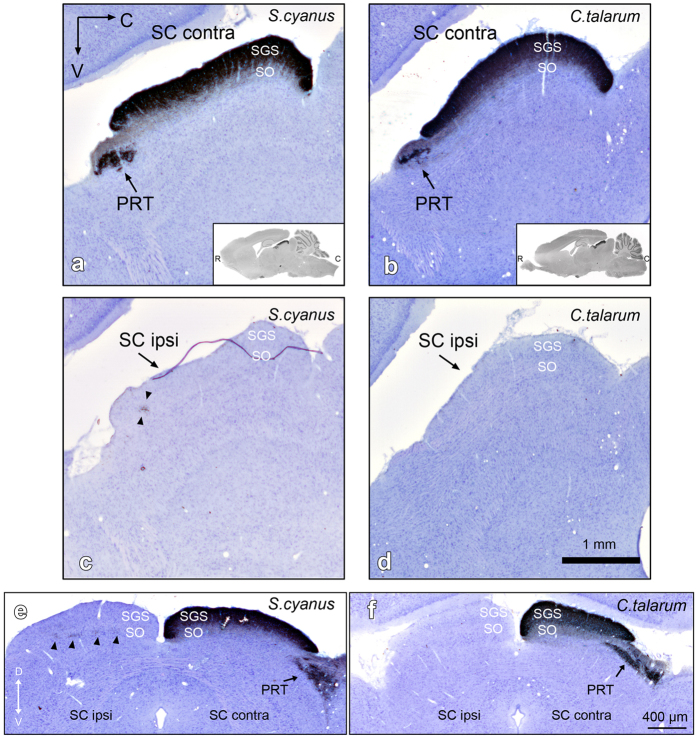
Sagittal and coronal sections showing CTB-labeled retinal fibers in the contra- and ipsilateral SC of *S. cyanus* (**a**,**c**,**e**) and *C. talarum* (**b**,**d**,**f**). Note that there are no labeled retinal fibers in the ipsilateral SC of *C. talarum*. Insets in (**a**,**b**) represent low magnification photomicrographs of the respective sagittal and coronal SC. Arrowheads in (**c**,**e**) indicate retinal terminals in the SO of *S. cyanus*. Counterstaining with Giemsa. R = rostral, C = caudal, V = ventral, D = dorsal.

**Figure 7:**
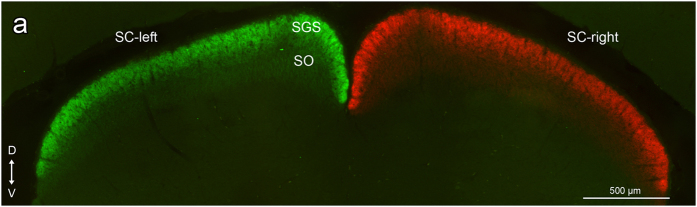
Transverse section showing CTB-Alexa labeled retinal fibers in the left (Alexa-488) and right (Alexa 546) superior colliculus of *C. talarum* (**a**). Note that there is no labeling of ipsilateral fibers in the SC, confirming the complete loss of this projection in the facultative non-strictly subterranean *C.talarum*. D = dorsal; V = ventral.

The SC receives massive retinal input in most mammals including rodents. This mesencephalic structure possesses two characteristic retinorecipient zones: the external stratum griseum superficiale (SGS), which has a dense plexus of retinal arborizations, and the internal stratum opticum (SO), which possesses less dense terminal arborizations^19^,^20^. Both the SGS and SO were clearly recognizable in our subterranean species. On the contralateral side, both zones appeared fully and uniformly covered with retinal terminals (as shown in Fig. 6a,b). On the contrary, the ipsilateral side of *S.cyanus’* SC showed discontinuous small patches of labeled terminals located close to each other (Fig. 6e), and restricted only to the dorsal portion of the rostral SO (Fig. 6c–e). The ipsilateral SC was devoid of labeled terminals in *C. talarum* (Figs 6d–f and 7a).

### Volumetric analysis of the GLd and SC

**Figure 8:**
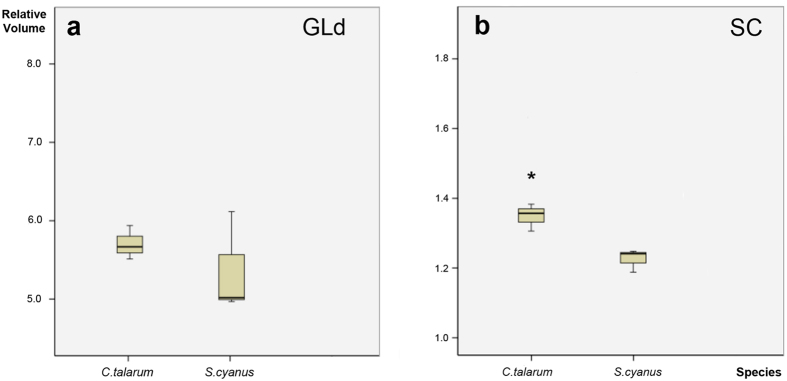
(**a**) Plot of the contralateral GLd volume relative to the brain volume in *C. talarum* and *S. cyanus*. No significant differences were observed between species (Kruskal-Wallis and Mann Whitney *U-*tests; *α* = 0.05; *P* > 0.05). Relative volume values are in multiples of 10^−7^. (**b**) Plot of the contralateral SC volume relative to the brain volume (Y axis). Asterisk indicates a significant difference between groups (Kruskal-Wallis and Mann Whitney *U-*tests; *α* = 0.05; *P* = 0.05). Relative volume values are in multiples of 10^−6^. The solid lines contained within the yellow boxes in (**a**,**b**) represents the mean.

The analysis of the contralateral projections showed that the overall volume occupied by retinal terminals within the GLd was not statistically different between *S. cyanus* and *C. talarum* (Fig. 8a). On the other hand, measurements of the retinal terminals in the SC indicated that the contralateral SC of *S. cyanus* had a smaller volume than that of *C. talarum* (Fig. 8b).

### Projections to other retinorecipient nuclei

To assess whether the decrease in the ipsilateral projection volume in the GLd and SC of *C. talarum* applies to other retinorecipient structures, we decided to measure the volume of retinal terminals in two retinorecipient structures not directly involved with the thalamo- and tectofugal projections. These nuclei were the medial terminal nucleus (MTN) and suprachiasmatic nucleus (SCN). The MTN provides visual inputs to neural circuits implicated in visuomotor reflexes such as the optokinetic nystagmus^21^, whereas the SCN is the main central pacemaker for circadian rhythms^22^.

In *S. cyanus* and *C. talarum* retinal projections to the MTN were observed only in the contralateral hemisphere. These terminals formed a dense plexus of fine endings distributed evenly throughout the whole nucleus. Conversely, the retinal projections to the SCN were bilateral, and covered the nuclei evenly on both sides. These terminals showed a distinctive plexus of less dense endings. While the volumetric measurements for the MTN retinal terminals were not significantly different between *S. cyanus* and *C. talarum*, the volume of the SCN was significantly larger in *C. talarum (P* ≤ 0.05; data not shown).

## Discussion

Although it seems that visual conditions inside burrows could be similar to those of nocturnal/dim-light environments, there is no evidence in current literature indicating nocturnal traits in the visual system of subterranean species. In this regard, previous studies show a retinal cone proportion of 10% for *S. cyanus*^9^, 10–14% for *C. talarum*^10^, 10–30% for *Ctenomys magallanicus*^10^ and 26% for the pocket gopher *Thomomys bottae*^23^. All these values are similar to the ones reported for diurnal surface-dwelling rodents such as *Octodon degus* (30%)^24^ and the agouti *Dasyprocta aguti* (10%)^25^. Also, subterranean rodents such as *C. talarum, C. magallanicus, Fukomys anselli, Fukomys mechowii* and *S. cyanus* have fewer rods than the typical mammalian average^9^,^10^,^11^ and far less than nocturnal species^26^. In addition, radio-telemetric experiments on free-living *S. cyanus* and *C. talarum* showed that its daily activity is generally concentrated in the diurnal period^27^,^28^. Nevertheless, coruros kept in the laboratory show nocturnal activity^29^. Lastly, recent behavioral experiments indicate that visual capabilities of *S. cyanus* are comparable to that of diurnal surface-dwelling rodents^30^.

Our results confirm and extend the aforementioned observations, as they show that *S. cyanus* and *C. talarum* have non-regressive visual systems, whose characteristics resemble more their diurnal than nocturnal close relatives. The overall values measured for the visual field size and binocular overlap in these two species were comparable to the values found in their diurnal/crepuscular close relative, *Octodon degus*^15^, and very similar to those exhibited by other small mammals (Muridae, Sciuridae)^31^. The density and distribution of cells in the ganglion cell layer, featuring a well-developed visual streak in both species, are also comparable to those exhibited by *O. degus*^15^ and the agouti^32^. The visual central projections follow the expected pattern for typical small mammals^19^,^20^, indicating a well-developed but unspecialized visual system. Conversely, we did not find in these species any of the features that characterize the visual system of their crepuscular/nocturnal close relative *O. lunatus*^15^, namely, an increase in binocular overlap, a comparatively low number of ganglion cells, and a relative increase in the ipsilateral retinal projections to the dorsal thalamus (Table 1).

A recent study^33^ showed that light levels inside the burrows could match scotopic conditions even dozens of meters away from the entrance, as long as galleries follow a straight course. However, most subterranean animals build sinuous and intricate gallery systems, and usually keep the burrow entrances obstructed by soil plugs, which extremely hinders both light availability and effective light propagation inside the burrows. Altogether, our results and the previous observations indicate that subterranean ecotopes differ substantially from nocturnal ones. According to this evidence, subterranean animals do not live under moonlight-like illumination, as nocturnal animals do. Rather, these animals seem to live either in total darkness, which renders vision useless, or in alternating episodes of daylight and darkness, which seems to correlate with the preservation of diurnal features in their visual system. A more thorough and comprehensive analysis of the visual ecology of subterranean mammals is needed to validate these conjectures (see below).

Another salient aspect of our results relates to the binocular visual attributes exhibited by these species. Stereopsis, as well as other significant dimensions of visual experience such as enhanced contrast and light sensitivity, rely crucially on binocular fusion, i.e., the combination of the visual activity evoked from each eye in a unified and coherent perceptual dimension^34^,^35^. Binocular fusion can only occur in a region of binocular overlap of the visual field, and requires a precise alignment of the afferents arising from specular loci of each retina onto a common postsynaptic target. In mammals, binocular fusion depends critically on the apposition of ipsi- and contralateral retinal projections onto the GLd^17^,^36^,^37^. These ipsilateral projections arise from the area centralis and the retinal crescent located temporal to it. As a result, the domain of binocular fusion gets restricted to the frontal area of binocular superposition. The size and shape of this area vary greatly among mammals, being maximal in hominids, and very limited in ungulates with laterally positioned eyes^31^,^38^.

In addition to the frontal zone of binocular overlap, the visual field of rodents, as well as in many other mammals, features a dorso-medial zone of binocular overlap. In the case of octodontines, the blind area corresponding to the projection of the tip of the snout establishes a clear delimitation between these regions^15^. This dorsal binocular region mostly relates to the inferior/anterior retina, from where no ipsilateral projections to the GLd originate. Hence, this region cannot be a site of binocular fusion, nor can it be involved in stereopsis. The visual operations associated with the dorsal binocular overlap are still pending elucidation. It has been suggested that this area, in rodents, is mainly involved in surveillance for aerial predators, although some sort of binocular integration has also been hypothesized^39^. In this context, our results become highly relevant. *S. cyanus* has a definite zone of frontal binocular overlap and a corresponding temporally located area centralis, but also a highly reduced amount of ipsilateral retinal projections when compared with its diurnal relative *O. degus* (65% reduction). The case of *C. talarum* is even more extreme, as it lacks a fronto-ventral area of binocular superposition, has no recognizable area centralis in the temporal retina, and shows no ipsilateral retinal projections. On the other hand, in both species the shape and extension of the monocular visual field and the dorsal region of binocular overlap, as well as the whole set of contralateral visual projections, appear well-developed up to a degree comparable to *O. degus*^15^.

Thus, our results strongly suggest that the adoption of a subterranean lifestyle lead, in these rodents, to an unexpected and highly unconventional visual regression associated specifically with the neural structures involved in binocular fusion. This trend is strikingly more pronounced in *C. talarum* than in *S. cyanus*, possibly reflecting the elapsed time since each species adopted the subterranean lifestyle. Fossil and molecular data indicate that the origin of *S. cyanus* and *Ctenomys* date 2–5 Ma^40^,^41^,^42^ and at least 3.6 Ma ago^43^,^44^, respectively. In addition, the ancestor of *S. cyanus* has been suggested to be a surface-dwelling degus-like rodent^40^, while the putative ancestor of *C. talarum* may have subterranean habits and moderate digging specializations^44^.

How is it possible that only visual traits underlying binocular fusion had suffer a significant regression? Since the aforementioned finding was unexpected to us, and to our knowledge there is no precedent in literature, we cannot provide a definitive answer to this question at this point. We may however, outline a conceptual scenario that enables us to elaborate on such question. As we stated above, in mammals, binocular fusion takes place only within the frontal binocular field. This fact points towards a close association between binocular fusion and what we would call “active vision”, i.e., the repertoire of behaviors through which animals act upon their environment such as grasping, climbing, manipulating and biting. Indeed, besides nocturnal habits, several forms of active vision such as predatory habits^34^,^45^,^46^ and eye/hand coordination^47^,^48^ have been suggested to be major evolutionary factors leading to the enhancement of binocular integration in mammals as well as in birds^49^. On the other hand, the monocular “panoramic” visual field as well as the dorsal area of binocular superposition seems to be associated with what can be denoted as “reactive vision”. That is, reactive/defensive behaviors triggered by or oriented towards the detection of salient/emerging environmental features such as visual surveillance, escape reactions and predator detection^38^,^50^. Highly relevant in this context is the recent finding that eye movements of freely moving rats tend to maximize the degree of dorsal binocular superposition at the expense of frontal superposition^39^. This finding is compatible with the dorsal-area’s proposed role in surveillance against aerial predators.

Thus, our results support the view that a subterranean lifestyle entails a loss of active vision, but the maintenance of the reactive vision. This viewpoint is indeed in agreement with the visual ecology of these species. In fact, facultative non-strictly subterranean animals behave in a dual fashion: they spend most of their daily activities in scotopic/lightless underground burrows, but also exhibit significant photopic activity above ground. On the surface, these animals display surveillance at the entrance or near the burrows, fast displacement between burrows, and occasionally short-range migrations to neighboring areas, where they rush to build a new burrow system^2^,^28^,^51^,^52^. For all these activities reactive vision is indispensable, as the animals are highly exposed to aerial and surface-dwelling predators, namely foxes, diurnal owls and birds of prey^53^. By contrast, all other activities that may require active vision such as foraging and feeding, conspecific recognition, courtship, offspring parental care and digging, takes place mainly in the subterranean ecotope^28^,^51^, an environment that does not favor visual operations. Thus, we propose that diurnal surface life is responsible for the maintenance of the reactive visual dimension, while the underground life underlies the loss of the active visual dimension. A broader and detailed comparative analysis is indeed required to validate this hypothesis.

**Figure 9:**
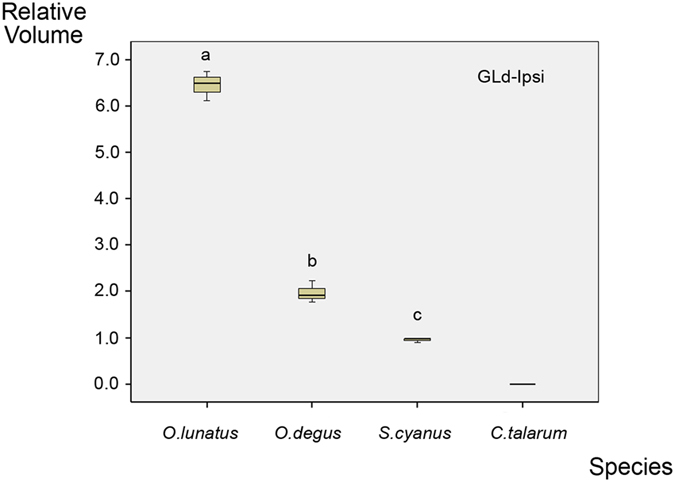
Plot of the ipsilateral GLd volume relative to the brain volume in *O. lunatus* (nocturnal, surface-dwelling), *O. degus* (diurnal, surface-dwelling), *S. cyanus* (diurnal, subterranean) and *C. talarum* (diurnal, subterranean). Differences between groups are significant (Kruskal-Wallis and Mann Whitney *U-*tests; *α* = 0.05; *P* = 0.05). Note that the value for *C. talarum* is 0. Relative volume values are in multiples of 10^−8^. The solid lines contained within the yellow boxes represent the mean.

Finally, we would like to emphasize that among vertebrates, sensory systems have remarkable plasticity, both in ontogeny and phylogeny. This plasticity allows for these animals an almost infinite variety of different lifestyles and ecological specializations. Thus, the question of how and to which extent different features of the sensory systems become shaped by particularities of the sensory behaviors entailed in the living of different organisms is worth to be studied. A direct approach is to compare the sensory specializations of phylogenetically closely related organisms that exhibit significant differences in their way of life. Here we investigated some significant architectural traits of different structural components of the visual system (visual field organization, ganglion cell layer distribution, retinal projections organization) in two species of subterranean octodontoids, aiming to compare these traits with that of phylogenetically closely related species that differ in visual habits (Table 1, Fig. 9, S1). The results of our study were, above all, unexpected, as they show that subterranean octodontoids exhibit a unique regression of a suite of traits, all of which are operationally linked with stereopsis and binocular fusion. We conjecture that this outcome is the result of the adoption of a lifestyle that emphasizes the reactive dimensions of visual behavior over the active ones, a conjecture whose validity requires further and more detailed comparative studies. Strikingly, our results suggest that the shaping of the visual system by behavior seems to have a “surgical quality”, in as much as it is very precise, direct and profound. The mechanisms underlying this process deserve, at the very least, to be more and better studied.

## Methods

Three *Spalacopus cyanus* and six *Ctenomys talarum* weighing 120–160 g and including both adult males and females were captured in the wild and kept in standard animal cages. Specifically, *S.cyanus* was captured in Los Molles (Región de Valparaiso, Chile) and *C.talarum* in Mar de Cobo (Provincia de Buenos Aires, Argentina). Animals were maintained in an animal facility at the University of Chile. All animals were treated following the protocols of the National Institute of Health Guide for the Care and Use of Laboratory animals, and the guidelines Animal Ethics Committee of the University of Chile. The experimental procedures were approved by the Faculty of Sciences of University of Chile Ethics Committee (Permit 29–9–011). All efforts were made to minimize animal numbers and suffering.

### Visual field measurements

Measurements of visual fields were taken in two individuals per species. Animals were anesthetized with a mixture of ketamine (120 mg/kg IP) and xylazine (4 mg/kg IP) and mounted in a stereotaxic head holder inside a campimeter. The head was held in position at the centre of the visual perimeter so that the palpebral fissures coincided with 0° on the rotation axis of the campimeter. The perimeter coordinates followed the conventional latitude and longitude system. This coordinate system was used for the presentation of visual field data (Fig. 1). The eyes were examined using an ophthalmoscope reflex technique. For each eye the visual fields were determined by measuring the limits of the nasal and temporal reflected retinas. To avoid ocular movements during the measurements, eyes were paralyzed locally with intraorbital injections of Lidocaine.

### Preparation of retinal whole mounts and ganglion cell layer counts

Following fixation (transcardial perfusion, see below), palpebral fissures and dorsal sclera were marked onto the eye balls with micro-sutures for orientation. The eyes were then enucleated and washed in 0.1 M PBS. Retinas were carefully dissected from their underlying pigmented layer and the optic nerves were severed just beneath their retinal attachment. The isolated retinas were washed again in PBS and mounted on moist gelatinized slides. Once defatted with chloroform, retinas were stained with 1% cresyl violet for four minutes, and thereafter dehydrated through an ascending alcohol series, cleared in xylene and cover-slipped with permount.

Following Pettigrew^54^, two whole-mounted retinas for each species were drawn on a sheet of graph paper using an overhead projector. Care was taken to align the sides of the microscope slide to the graph paper grid. The X-Y coordinates of a number of landmarks in the retinas were noted in the drawing so that all subsequent counts could be transferred to the drawing from the stage micrometer. At 400× magnification, cells in the ganglion cell layer were counted in a 125 × 125 μm optic grid. We counted neurons in the ganglion cell layer according to the fractionator principle^55^. Counts were taken at 1.0 mm intervals across the whole retina, except for the high density area centralis region, in which counts were taken at 0.5 mm intervals. These counts were converted to densities (ganglion cells layer neurons/mm^2^). Plots of isodensity contours overlaying the photograph of the corresponding retina were prepared using a previously published R script for the construction of topographic maps of retinal cell density (for details see Garza-Gisholt *et al*.^56^). The total ganglion cell layer population was estimated by using the mean cell densities for each of the isodensity curves (except for the area centralis where we used the values of the contouring AC isodensity curves and multiplied those values by the respective areas (in mm^2^), see earlier studies for details^9^,^45^. Because it was not possible to discriminate between ganglion cells and displaced amacrine cells based on cytological criteria, cell counts presented in this paper always include both cell types.

### Labeling from retinal projections

#### Unilateral injections

Six animals (three *S. cyanus* and three *C. talarum*) were anesthetized as previously mentioned, and given intraocular injections (8 μL, left eye) of 0.5% Cholera toxin subunit B (CTB, List Biological Laboratories) mixed with 1% DMSO. After a seven-day survival period, animals were deeply anesthetized with ketamine (120 mg/kg IP) and xylazine (4 mg/kg IP), and perfused transcardially with approximately 500 mL of saline, followed by 500 mL of 4% paraformaldehyde in PB (pH 7.4). After removal of the skull, each brain was postfixed overnight in the same fixative and then transferred into a solution of 30% sucrose/PB until it sank. The brains were cut in coronal, sagittal and horizontal planes at a thickness of 30 μm and processed using immunohistochemical techniques to reveal CTB distribution with the avidin-biotin-peroxidase method. Briefly, sections were incubated with antibodies against CTB (made in goat, diluted 1:20,000), followed by biotinylated secondary antibodies (anti-goat IgG made in rabbit, diluted 1:200; Vector Laboratories). Avidin-coupled peroxidase (Vector ABC kit) was then used with diaminobenzidine as the final chromogen. A one-in-four series from sections was examined for CTB and was later counterstained with Giemsa to reveal cell bodies. A separate one-in-four series from sections was left without counterstaining. A third series from sections was stained with cresyl violet.

#### Bilateral injections

Additionally, three *C. talarum* were used to confirm the absence of ipsilateral projections. Animals were deeply anesthetized with ketamine (120 mg/kg IP) and xylazine (4 mg/kg IP) and received 8 μL of 0.5% CTB-Alexa-488 (Molecular Probes) into the right eye, and the same volume of CTB-Alexa-594 (Molecular Probes) into the left eye. The processing of the animals was realized as aforementioned. After removal of the skull, each brain was postfixed overnight in the same fixative and then transferred into a solution of 30% sucrose/PB until it sank. The processing of the animals was the same as described above. Sections were coverslipped with PB for visualization of the fluorescence.

#### Relative volume from retinal terminals

Volume measurements were taken using the CTB-Giemsa series. For this purpose we took high-resolution images with a scanning CCD camera (Leaf System, Inc., Southborough, MA) equipped with a 120 mm macro lens. Image processing was done in Adobe Photoshop CS5. The areas of entire selected nuclei were measured across transverse, sagittal and horizontal sections. Volumes were calculated by multiplying the area in each section by the thickness of the section (30 μm) and the sampling interval (120 μm) using the Volumest plugin^57^ in ImageJ. We measured the contralateral/ipsilateral retinal labeling terminals volume of the GLd and SC, and the contralateral retinal labeling terminals volume of the suprachiasmatic nucleus (SCN) and of the medial terminal nucleus (MTN). To account for allometric effects, we divided the volume of a given structure by the total brain volume. Brains were therefore weighed to the nearest milligram and brain volume was subsequently calculated by dividing brain mass by the fixed brain tissue density (1.036 g/mm^3^)^58^. Thus, volumes of the measured structures are expressed as values relative to the brain volume.

#### Statistical analysis of retinal terminals volumes

Due to our small sample size, we used non-parametric Kruskal-Wallis and Mann Whitney *U-*tests to compare volumes between species. The α-level for all tests was set to 0.05.

## Additional Information

**How to cite this article:** Vega-Zuniga, T. *et al*. Selective binocular vision loss in two subterranean caviomorph rodents: *Spalacopus cyanus* and *Ctenomys talarum. Sci. Rep.* **7**, 41704; doi: 10.1038/srep41704 (2017).

**Publisher's note:** Springer Nature remains neutral with regard to jurisdictional claims in published maps and institutional affiliations.

## Supplementary Material

Supplementary Figure S1

## Figures and Tables

**Table 1:** Summary table that compares data from *S. cyanus, C. talarum, O. degus* and *O. lunatus*.

Species	Lifestyle	Binocular overlap	Peak density	Overall density	Total estimation	Ipsilateral projection to GLd	Ipsilateral projection to SC
*S. cyanus*	Diurnal subterranean	50°	6,400	3,321	83,936	1.76%	Traces
		53°	6,592	3,358	84,131		
*C. talarum*	Diurnal subterranean	58°	7,744	3,407	102,482	No	No
		60°	7,872	3,534	103,874		
O. degus*	Diurnal Surface dwelling	50°	6,384	2,990	180,000	2.75%	0.16%
O. lunatus*	Nocturnal Surface dwelling	100°	4,352	1,638	108,000	10.52%	0.95%

Peak density and overall density corresponds to cells located in the retinal ganglion cell layer. Total estimation was calculated subtracting the value for displaced amacrine cells present in the ganglion cell layer. For *S. cyanus* and *C. talarum* we show data of two individuals per species. ^*^Data for *O. degus* and *O. lunatus* obtained from Vega-Zuniga *et al*.^15^.
