# Supplementary figures and images for: Selective binocular vision loss in two subterranean caviomorph rodents: Spalacopus cyanus and Ctenomys talarum
Source: Sci Rep. 2017 Feb 2;7:41704. doi: 10.1038/srep41704 (PMC5288697; doi:10.1038/srep41704)

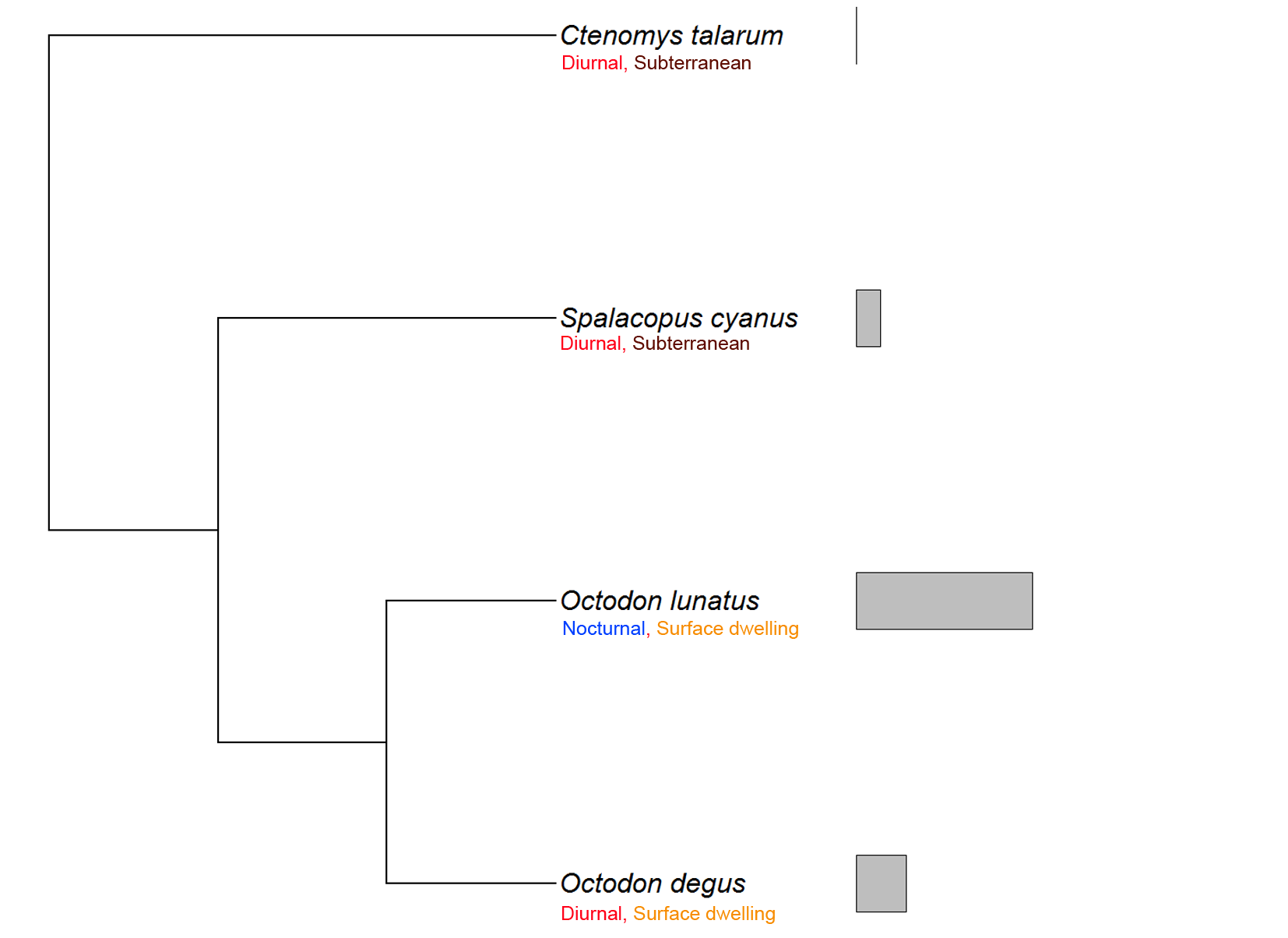

Supplement: Supplementary Figure S1 [file srep41704-s1.tiff]
